# Adenovirus 36 Attenuates Weight Loss from Exercise but Improves Glycemic Control by Increasing Mitochondrial Activity in the Liver

**DOI:** 10.1371/journal.pone.0114534

**Published:** 2014-12-05

**Authors:** Ha-Na Na, Young-Mi Hong, Michael B. Ye, Sooho Park, In-Beom Kim, Jae-Hwan Nam

**Affiliations:** 1 Department of Biotechnology, The Catholic University of Korea, Bucheon, Republic of Korea; 2 Department of Pediatrics, Ewha Womans University Medical School, Seoul, Republic of Korea; 3 Division of Liberal Arts and Sciences, Gwangju Institute of Science and Technology, Gwangju, Republic of Korea; 4 Department of Anatomy, College of Medicine, The Catholic University of Korea, Seoul, Republic of Korea; University of Regensburg, Germany

## Abstract

Human adenovirus type 36 (Ad36) as an obesity agent induces adiposity by increasing glucose uptake and promoting chronic inflammation in fat tissues; in contrast, exercise reduces total body fat and inflammation. Our objective was to determine the association between Ad36 and the effects of exercise on inflammation and glycemic control. In the human trials (n = 54), Korean children (aged 12–14 years) exercised for 60 min on three occasions each week for 2 months. We compared the body mass index (BMI) Z-scores before and after exercise. C57BL/6 mice were infected with Ad36 and Ad2 as a control, and these mice exercised for 12 weeks postinfection. After the exercise period, we determined the serum parameters and assessed the presence of inflammation and the mitochondrial function in the organs. Ad36-seropositive children who were subjected to a supervised exercise regimen had high BMI Z-scores whereas Ad36-seronegative children had lower scores. Similarly, Ad36-infected mice were resistant to weight loss and exhibited chronic inflammation of their adipose tissues despite frequent exercise. However, Ad36 combined with exercise reduced the levels of serum glucose, nonesterified fatty acids, total cholesterol, and insulin in virus-infected mice. Interestingly, virus infection increased the mitochondrial function in the liver, as demonstrated by the numbers of mitochondria, cytochrome c oxidase activity, and transcription of key mitochondrial genes. Therefore Ad36 counteracts the weight-loss effect of exercise and maintains the chronic inflammatory state, but glycemic control is improved by exercise synergistically because of increased mitochondrial activity in the liver.

## Introduction

Obesity, a disease closely related to other metabolic disorders [1], has broad etiology including diet, physical activity, genetics, and exposure to pathogens [2]–[4]. Among the pathogen-associated causes, adenovirus 36 (Ad36) is an important factor in human and animal adiposity [3]–[6], perhaps 1) by promoting differentiation of stem cells into adipocytes, 2) by differentiating preadipocytes into adipocytes, and/or 3) by stimulating lipid accumulation by increasing glucose uptake in adipocytes [7]–[9]. Although direct evidence proving that Ad36 infection of humans triggers obesity is lacking due to the ethical issues associated with human experimentation, studies from the USA, Italy, and Korea have demonstrated that Ad36 seropositivity is correlated with obesity [5], [10], [11], [12]. Importantly, although Ad36 increases adiposity, the virus can improve glycemic control as blood lipids and insulin levels decrease [13.14].

Some studies have demonstrated that inflammation is related to weight-gain stimulation and increased adiposity, which subsequently leads to the development of metabolic syndrome [15]–[18]. In addition, there is an interesting link between obesity and pathogens because the pathogen burden is associated with an increased fat mass in humans and low-grade chronic inflammation [19].

Recently, we reported that Ad36-induced obesity is associated with chronic inflammation based on the elevated levels of monocyte chemotactic protein-1 (MCP-1), a chemokine that recruits immune cells and causes macrophage infiltration of adipocytes [20]. We also found that MCP-1 is a prerequisite cellular factor for Ad36-induced adiposity because *MCP-1* knockout (*MCP-1^–/–^*) mice do not exhibit any induction of adiposity after virus infection [20]. Overall, these observations led us to hypothesize that Ad36, an inducer of chronic inflammation and adiposity, blocks the beneficial effects of exercise, such as reduced fat mass and lower inflammation [21]–[24].

Although Ad36 triggers an inflammatory response, this does not involve any metabolic variables such as blood glucose and free fatty acids in Ad36-infected mice [20]. Similarly, Ad36 improves hyperglycemia and hepatic steatosis in high-fat diet mice, and there is also evidence of reduced hyperglycemia and lower lipid concentrations in the livers of Ad36-seropositive humans [23] It has been proposed that Ad36 infection increases glucose uptake in adipocytes by activating the Ras and Akt signaling pathways, thereby leading to increased translocation of Glut4 (glucose transporter 4) [7], [8], [25]. This mechanism could explain the Ad36-related paradox of increased adiposity, but decreased metabolic variables. However, other mechanisms may also be involved. For example, we observed that Ad36 increases the expression of some genes that are involved in mitochondrial signaling pathways [9]. Mitochondrial dysfunction (defined as reduced oxidation and adenosine triphosphate synthesis) plays a major role in the etiology of obesity and type 2 diabetes mellitus [26], [27]. In addition, exercise-induced mitochondrial biosynthesis stimulates glucose uptake in skeletal muscles [28] Therefore, we also hypothesize that Ad36 stimulates the activity of mitochondria and that this leads to reduced blood glucose concentrations, which is similar to the effects of exercise.

In this study, we aimed to determine the effects of Ad36 on weight loss caused by exercise and on improved glycemic control due to increased mitochondrial activity. Understanding the underlying mechanism and the roles of Ad36 infection-induced phenotypes, such as obesity and inflammation, may provide insights into the etiology of different types of obesity.

## Materials and Methods

### Ethics statement

The human study was part of the Ewha Womans University Medical Center (EWUMC) Obesity Research Program. Informed written consent for participation was obtained from each individual and their legal guardians, and the study design was approved by the Institutional Ethics Review Board at the EWUMC (Protocol ID. ECT 11-11-16).

All mice were handled according to the guidelines and regulations of the Korean Association for Laboratory Animals. The protocol was approved by the International Animal Care and Use Committee at Sungsim Campus, The Catholic University of Korea (#2012-017).

### Experimental design of the human exercise program

In total, 54 schoolchildren (45 boys and 9 girls) aged 12–14 years were recruited from middle schools in Seoul, Korea. The subjects were in the ≥95th percentile for body mass index (BMI) on the Korean reference BMI-for-age curves, according to the definitions of the International Obesity Task Force. The exercise protocol comprised an aerobics program and a complex program (circuit weight training [CWT] and aerobic exercise, Table S1). The subjects were divided into three groups: aerobics program (*n* = 16), complex program (*n* = 20), and control (no exercise, *n* = 18) [29]. In the aerobics program, which was based on a program from the American College of Sports Medicine (ACSM) (2000), the subjects exercised three times each week (twice under the supervision of a trainer and once independently) for 60 min per day on each occasion. The 60–80% maximal oxygen consumption (VO_2 max_) and 70–90% maximum heart rate (HR_max_) were obtained from graded exercise test data and were used to estimate the time required to elicit an energy expenditure of 300–400 kcal. After stretching and jogging for 5 min, the subjects exercised according to the main program (including soccer, basketball, and aerobics) for 50 min and stretched for 5 min. The complex program was based on CWT and ACSM (2000), and the subjects performed this program three times each week (two times CWT session with a trainer and one time independent aerobic exercise session) for 60 min on each occasion. It consisted of a warm-up, main, and cool-down exercises. The subjects were guided to stretch and jog for 5 min during the warm-up exercise. The main exercise included CWT and aerobic exercise, and the subjects were directed to exercise for 30 sec with 10 sec rest intervals. The subjects stretched for 5 min during the cool-down exercise. The 70–80% maximum strength levels were obtained from graded exercise test data. After stretching and jogging for 5 min, the subjects exercised according to the main program (CWT) for 50 min and stretched for 5 min.

### Experimental design of the mouse exercise program

Four-week-old female C57BL/6 mice were purchased from the Orient Company (Sungnam, Korea). The mice were adapted to laboratory conditions (18–23°C, 55–60% humidity, and 12∶12 h light∶dark cycle with the lights on at 07:00) for at least 1 week before the study. The mice were fed a normal diet and water was provided ad libitum. The mice were divided into six groups: nonexercise sham group, exercise sham group, nonexercise Ad2-infected group, exercise Ad2-infected group, nonexercise Ad36-infected group, and exercise Ad36-infected group. The mice were injected intraperitoneally with Ad36 at 5×10^6^ plaque-forming units (pfu) per mouse or Ad2 (5×10^6^ pfu per mouse), or with cell culture medium (sham treatment). Ad2 is a serotype C adenovirus and it has no association with human obesity [30]. Thus, many previous studies have used Ad2 as a negative control in virus-induced obesity experiments [30]–[32].

The mice were subjected to exercise and infection simultaneously. Individual mice were placed onto treadmills and exercised by running for 30 min per day (5 days per week). Each animal ran at the same speed each day (average of 17 m per min). This exercise program was designed as a validation study (using different mice) where the sham-, Ad2-, and Ad36-infected mice (*n* = 8 per group) were given free access to treadmills over the course of 12 weeks. The exercise sessions were conducted during the light phase.

The body weight and food intake of the mice were measured weekly throughout the study. At 12 weeks after infection, the mice were fasted overnight and sacrificed, and their blood was collected by cardiac puncture. The epidermal fat pads were weighed and the average values were recorded. Blood samples were collected and centrifuged at 4°C to separate the plasma. The plasma and organs were stored at −80°C until analysis.

### Gene expression

Total RNAs were isolated from the epidermal fat pads, livers, and muscle using TRIzol Reagent (Invitrogen, NY). The complementary DNA was synthesized from the total RNA using RT & Go (MP Biomedicals, Santa Ana, CA) with an oligo(dT) primer (Cosmo Genetech, Korea) and reverse transcription. Real-time PCR was performed on a MyiQ single-color real-time PCR cycler (Bio-Rad, CA) using the SYBR Green PCR Master Mix (Takara Bio Inc., Japan). The samples were analyzed in duplicate, and each transcript level was adjusted to that of a housekeeping gene (18S rRNA). The expression of each gene was quantified and expressed as an mRNA level relative to that of the control gene, i.e., after normalization to 18S rRNA, using a 2^–ΔΔCT^ formula. We checked the expression of inflammation-associated genes (encoding MCP-1, TNF-α, F4/80, CD64, and CD206), and mitochondrial genes (encoding PGC-1α, NRF-1, mtDNA, and UCP-1).

### Enzyme-linked immunosorbent assay

Proteins were extracted from epidermal fat pads after tissue homogenization and separation of the supernatant by centrifugation. The concentration of MCP-1 in the adipose tissues of the mice was measured using a Quantikine Mouse CCL2/JE/MCP-1 Immunoassay kit (R&D Systems, MN), according to the manufacturer's instructions. The concentration of TNF-α was measured using a Ready-SET-Go ELISA kit (eBioscience, CA).

### Flow cytometry

Epidermal fat pads were chopped in Dulbecco's modified Eagle's medium containing 5% fetal bovine serum (FBS) and 4 mg/mL type I collagenase, and digested at 37°C for 30 min. Three volumes of PBS (with 5% FBS) were added to the fat pads. The digested fat pads were filtered through 100 µm nylon mesh and the filtered fraction was centrifuged at 200×*g* to isolate the stromal vascular fraction (SVF) from the fat pads. The red blood cells in the SVF were lysed with Pharm Lyse (BD, NJ) and incubated with Mouse Fc Block (BD Pharmingen purified rat anti-mouse CD16/CD32) for 20 min. The macrophages in the SVF were counted and incubated with 0.2 µg of rabbit anti-F4/80 antibody and isotype matched control antibody (Santa Cruz Biotechnology, TX) for 30 min at 4°C. The cells were washed with PBS and incubated with fluorescein-isothiocyanate-labeled anti-rabbit antibody (AbCam, MA) for 30 min, washed twice, and fixed in 2% paraformaldehyde. A FACScan flow cytometer (Beckman FC500) was used to record 10,000 events.

### Adipose tissue histology

Epidermal fat pads were fixed in 1% paraformaldehyde (Sigma, MO) for 12–16 h at 4°C and embedded in paraffin. Sections (5 µm thick) were cut at 50 µm intervals, mounted on charged glass slides, and stained with hematoxylin and eosin to identify the infiltrating immune cells. Samples of the fat pads were visualized under a microscope and photographed with AxioVision version 4.8 software (Carl Zeiss, Germany).

### Assay of serum parameters

The sera of mice were collected by cardiac puncture. The concentrations of total cholesterol, triglyceride, neutral free fatty acids, high density lipase–cholesterol, and low density lipase–cholesterol were measured using a COBAS Integra 800 analyzer. Plasma insulin concentrations were measured using the insulin (mouse) ELISA kit (80-INSMS-E01, ALPCO Diagnostics, NH).

### Immunoblotting

Liver and muscle were homogenized in lysis buffer (50 mM Tris-HCl [pH 8.0], 150 mM NaCl, 5 mM EDTA, 1% NP-40, protease inhibitor cocktail, and phenylmethanesulfonyl fluoride). The protein concentrations in the tissue extracts were determined using a Bradford protein assay (Bio-Rad, CA). Tissue proteins (30 µg) were separated by SDS–PAGE in SDS electrophoresis buffer, transferred to a nitrocellulose membrane, and probed overnight with antibodies directed against p-AMPK (1∶1000; Cell Signaling, MA), and actin (1∶500; Santa Cruz Biotechnology, TX). The proteins were visualized with horseradish-peroxidase-conjugated to anti-immunoglobulin G antibody and enhanced chemiluminescence (eBioscience, CA).

### Mitochondria isolation and activity

The mice were starved overnight before isolating mitochondria experiment. The mitochondria (100 mg) in the liver and muscle tissues were isolated by differential centrifugation. The animals were sacrificed and the livers were rapidly removed from the peritoneal cavity, and immersed in 50 mL of ice-cold extraction buffer A (10 mM HEPES [pH 7.5] containing 200 mM mannitol, 70 mM sucrose, and 1 mM EGTA). The livers were rinsed of blood with ice-cold extraction buffer A. The livers were minced with scissors and the extraction buffer was discarded and replaced with extraction buffer containing 2 mg/mL albumin. The livers were homogenized in a glass homogenizer with 3–4 strokes at 4°C. The homogenates were transferred to microcentrifuge tubes and centrifuged at 600×*g* for 5 min at 4°C. The supernatants were transferred to microcentrifuge tubes and centrifuged at 11,000×*g* for 10 min at 4°C. The supernatants were discarded and the pellets were washed with extraction buffer A. The supernatants were discarded and the pellets containing the mitochondria were resuspended and stored on ice.

The skeletal muscle was rapidly removed with a scalpel and immersed in a small beaker containing 5 mL of ice-cold extraction buffer B (20 mM MOPS [pH 7.5], containing 110 mM KCl and 1 mM EGTA). The muscles were minced with scissors and trimmed of visible fat, ligaments, and connective tissue. The minced muscles were washed twice with ice-cold extraction buffer B supplemented with 0.25 mg/mL trypsin. The minced muscles were resuspended in ice-cold extraction buffer B supplemented with 0.25 mg/mL trypsin for 20 min and centrifuged at 200×*g* for 5 min. The supernatant was discarded and the pellet was resuspended in extraction buffer B. The muscles were homogenized at 300×*g*, and the minced muscle was stroked 20 times. The supernatant was transferred to a microcentrifuge tube and centrifuged at 11,000×*g* for 10 min at 4°C. The supernatant was discarded, and the pellet was resuspended in ice-cold extraction buffer B and centrifuged at 11,000×*g* for 10 min at 4°C. The supernatant was discarded and the pellet containing the mitochondria resuspended.

The final mitochondrial pellets from the livers and skeletal muscle were each resuspended in 40 µL of storage buffer (10 mM HEPES [pH 7.4], containing 250 mM sucrose, 1 mM ATP, 0.08 mM ADP, 5 mM sodium succinate, 2 mM K_2_HPO_4_, and 1 mM DTT). The concentrations of mitochondrial proteins in the tissue extracts were determined using a Bradford protein assay (Bio-Rad, CA). Cytochrome c activity and mitochondrial membrane integrity were assessed as described by the manufacturer (Sigma, MO). To check cytochrome c activity, 0.95 ml of 1× assay buffer was placed in a cuvette to provide a baseline on a spectrophotometer. A suitable volume of enzyme solution or mitochondrial suspension was added to the cuvette and the reaction volume brought to 1.05 ml with 1× enzyme dilution buffer. The mitochondrial suspension was mixed by inversion. The reaction was started with the addition of 50 ml of ferrocytochrome c substrate solution and mixed by inversion. The reaction was assessed using the change of absorbance at 550 nm per minute as a result of the rapid reaction rate of this enzyme. The activity of the sample was calculated. To measure the outer membrane integrity of the mitochondria, two parallel samples of the mitochondrial suspension were diluted to 0.1 to 0.2 mg protein/ml with either 1× enzyme dilution buffer (cytochrome c oxidase activity in intact mitochondria) or with enzyme dilution buffer containing 1 mM n-dodecyl β-d-maltoside (total cytochrome c oxidase activity). The samples were incubated at 2–8°C for at least 10 minutes before assaying. Mitochondrial protein (1–2 mg) was taken and analyzed for cytochrome c oxidase activity. Activity in each sample was determined at ΔA_550nm_/minute, and the degree of mitochondrial integrity calculated.

### Electron microscopy (EM)

Transmission EM was used to determine mitochondrial characteristics as described previously. In brief, a small piece of liver, skeletal muscle, or inguinal fat sample was fixed in 1% osmium tetroxide, dehydrated with ethanol, and embedded in Spurr's resin. Thin sections (70 nm) were prepared using an ultramicrotome (Ultracut E; Reichert), placed on Cu/Pd grids (200 mesh size), and stained for 5 min in uranyl acetate, followed by 2 min in lead acetate. The preparation, fixation, and sectioning of all samples were performed by the EM facility at the Catholic University of Korea College of Medicine. Samples were viewed at 7500× magnification using a JEOL 1200EX transmission EM. Images were captured using an Advanced Microscopy Techniques XR-41 side-mount cooled, 4-megapixel transmission EM imaging system. Mitochondrial pathology was scored by observers blinded to the test sample treatments. Quantification of mitochondrion numbers and total area was performed using Adobe Photoshop.

### Statistical analysis

All data were analyzed using a Student's *t*-test in SAS software (v. 9.1; SAS Institute, Cary, NC, USA). The data were expressed as the mean ± standard error. Differences between means were considered significant at p<0.05. The data were analyzed using a one-way analysis of variance (ANOVA) to facilitate multiple comparisons.

## Results

### Effects of exercise on reductions of the total body mass and epidermal fat mass

**Figure 1:**
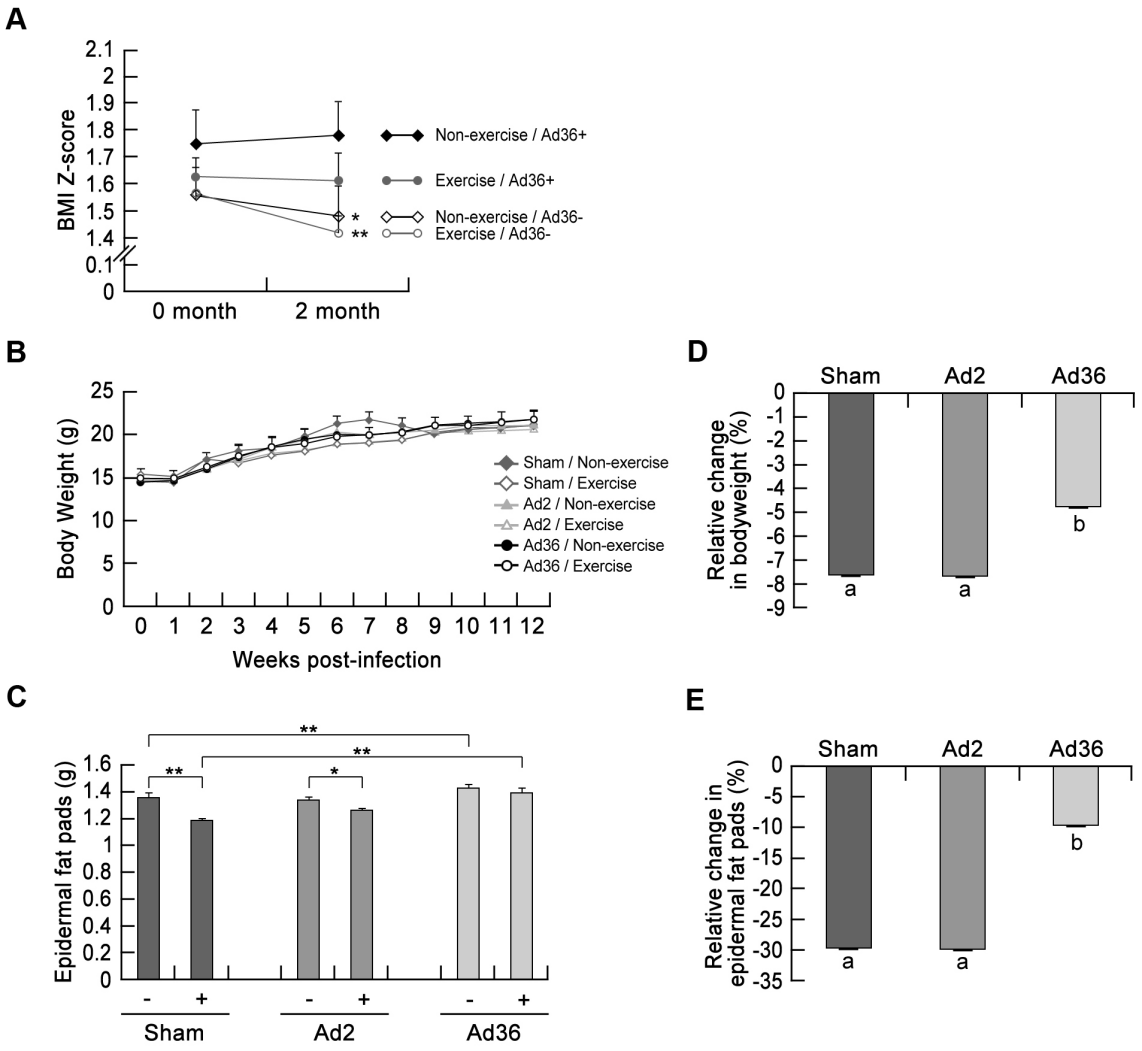
Reduction of the weight-loss effect of exercise by Ad36 infection. (A) Changes in human Z-scores body mass index (BMI) by Ad36 infection and exercise (*p<0.05, **p<0.01, compared with baseline (0 month) and at the end (2 months); *t*-test). (B) Mice were infected with Ad36 or Ad2 (virus control) or given sham injections (*n* = 8 per group). (C) The weights of the epidermal fat pads in mice were measured at 12 weeks post infection. The data are expressed as the mean ± standard error. (–, nonexercise mice; +, exercise mice; * p<0.05, ** p<0.01; *t*-test). (D, E) The relative changes in body weight (D; p<0.01, ANOVA) and epidermal fat pads (E; p<0.01; ANOVA) were calculated based on the mean difference between exercise and nonexercise mice at 12 weeks after infection. The data are expressed as the mean ± standard error.

Previous studies have shown that Ad36 infection increases the epidermal fat mass and inflammation in mice [20]. By contrast, exercise reduces both adiposity and inflammation [32], [33]. Therefore, we explored the relationship between Ad36-induced obesity and the beneficial effects of exercise in human and mice. In Ad36-seronegative children, the exercise regimen led to significant reductions in the BMI Z-scores (Figure 1A, exercise/Ad36–). However, there was no statistically significant decline in the BMI Z-scores of Ad36-seropositive children (Figure 1A, exercise/Ad36+). In agreement with previously published data [34], the Ad36-seropositive group had higher BMI Z-scores compared with the Ad36-seronegative group both initially and after 2 months of exercise (Figure 1A, Ad36+ vs Ad36–). Interestingly, over the course of 2 months, the rate of decline (slope) in the BMI Z-scores differed between groups (–0.018 for exercise/Ad36+ vs 0.0369 for nonexercise/Ad36+ and −0.179 for exercise/Ad36– vs −0.093 for nonexercise/Ad36–). Thus, our data demonstrate that the Ad36-seropositive children had difficulty losing weight by exercising.

Based on our demonstration that Ad36 counteracts the usual effects of exercise on weight loss in humans, we tested whether this also applies to mice. We compared the responses of Ad36-, Ad2- (negative control group), and sham-infected (inoculated with cell culture media) mice to normal diet and exercise. Each group was divided into two subgroups; one exercised on a treadmill for 12 weeks after ‘infection’ whereas the other did not. There were no differences in the food and water intake levels between groups and normal weight gains were observed in all groups (Figure 1B and Figure S1). We weighed the same mice after 12 weeks of infection/exercise in both the nonexercising and exercising groups. The difference between the groups after 12 weeks was normalized to the body weight of the nonexercising group and converted to a percentage. The calculated value means that the result for the exercising group represents the percentage of body weight based on the nonexercising group. In agreement with previous findings [20], the epidermal fat mass of the Ad36-infected group increased slightly compared with that of the sham group (Figure 1C). However, the relative changes in mean body mass and epidermal fat mass attributable to exercise were more remarkable. A comparison of the exercise and nonexercise groups revealed that the declines in body mass and epidermal fat mass were less pronounced in the Ad36-infected mice after 12 weeks of exercise (Figures 1D and 1E). Thus, the Ad36-infected mice appeared to be resistant to losses of their total body mass and epidermal fat mass even after exercising.

### Inflammation of adipocytes due to Ad36 infection

**Figure 2:**
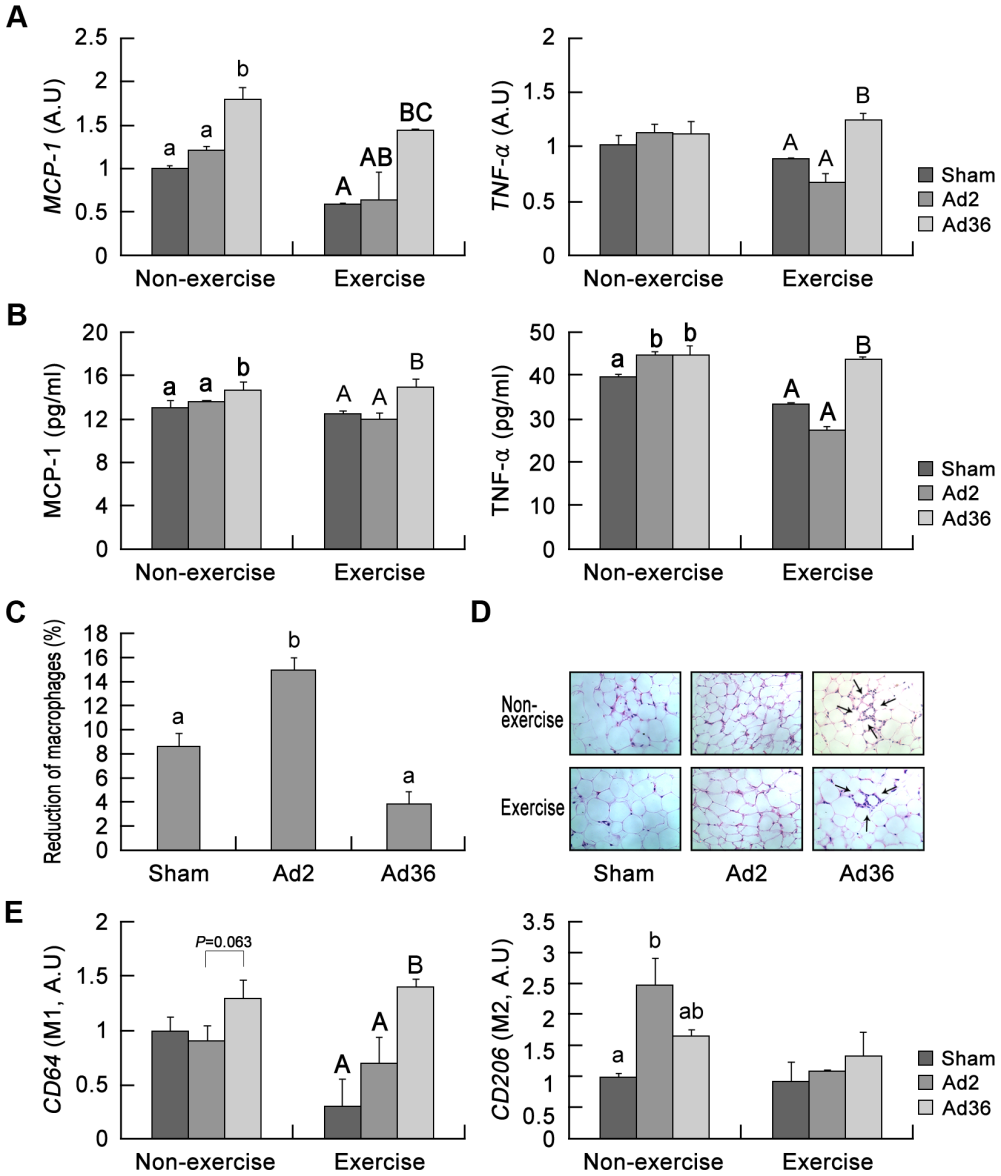
Maintenance of an inflammatory state by Ad36 infection despite exercise. (A) Mice were infected with Ad36 or Ad2, or given sham injections. The mRNA levels of inflammatory cytokines MCP-1 (p<0.05, ANOVA in nonexercise; p<0.001, ANOVA in exercise) and TNF-α (p<0.05, ANOVA) in the epidermal fat pads were assessed and the levels in exercise and nonexercise mice were compared with those in Ad36-infected mice. (B) The protein samples were obtained from the epidermal fat pads. The MCP-1 (p<0.05, ANOVA) and TNF-α (p<0.05, ANOVA in nonexercise; p<0.001, ANOVA in exercise) protein levels were assayed by ELISA. (C) Stromal vascular fractions were isolated from the epidermal fat pads and stained with F4/80 antibody as a macrophage marker. The relative reduction in macrophages was calculated based on the difference between nonexercise and exercise mice (p<0.05; ANOVA). (D) The epidermal fat pads were stained with hematoxylin and eosin (arrows indicate infiltrated immune cells). (E) The mRNA levels of macrophage markers were assessed (CD64 for M1 macrophages (p<0.05, ANOVA), CD206 for M2 macrophages (p<0.05, ANOVA), A.U., arbitrary units) to compare the levels in exercise and nonexercise Ad36-infected mice with those in sham-infected mice. The statistical analysis was carried out independently for each group, with lowercase letters indicating the nonexercising group, and capital letters for the exercising group. Statistically, groups with different letters over the bars were significantly different.

Recently, we reported that Ad36 requires MCP-1, a proinflammatory cytokine, to increase adiposity in mice [20]. In the present study, the mice that experienced an exercise regimen appeared to have lower levels of MCP-1 and tumor necrosis factor α (TNF-α) (both the protein and mRNA levels) compared with the nonexercise mice (Figures 2A and 2B), which indicates that exercise reduces inflammation. In addition, we determined the quantities and types of macrophages because M1 macrophages are specific indicators of an inflammatory state [20]. In agreement with previous data, macrophage infiltration, although generally reduced after exercise, was higher in the Ad36-infected mice than in the sham- and Ad2-infected mice according to flow cytometry and histological analysis of adipose tissues (Figures 2C and 2D). In addition, the M1 macrophage (CD64^+^) mRNA level in Ad2-or sham-infected mice was found to be lower than that in Ad36-infected mice after 12 weeks of exercise (Figure 2E). These results suggest that Ad36 can maintain inflammation within adipose tissues, even after exercise, which is considered to reduce inflammation.

### Synergetic effect of Ad36 infection and exercise on improved glycemic control

**Figure 3:**
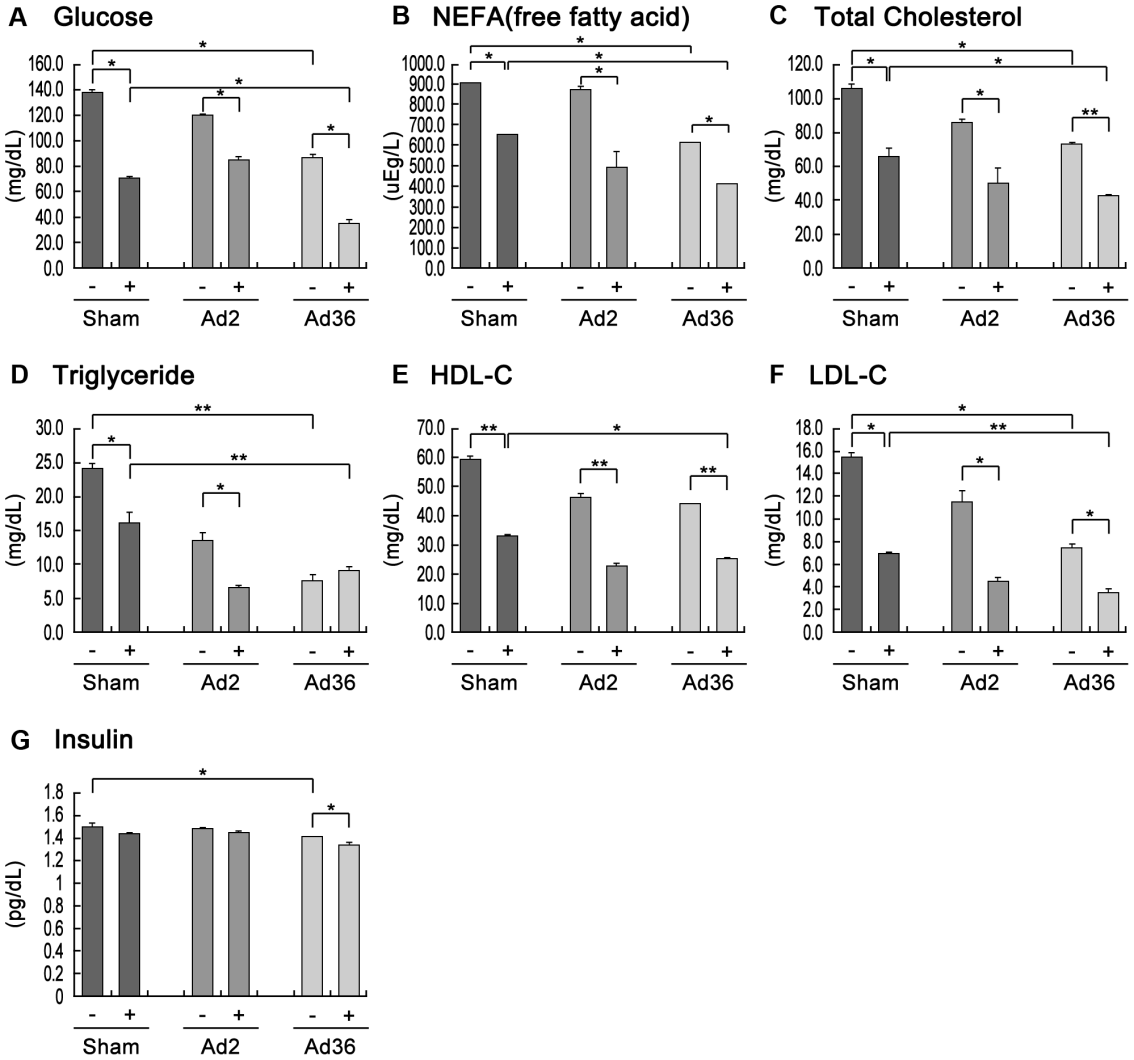
Reduction of the blood lipid levels due to the synergistic effect of Ad36 infection and exercise. Blood was obtained by cardiac puncture at 12 weeks after infection. The serum lipid parameters were measured using a COBAS INTEGRA 800 testing platform (Roche Diagnostics, Mannheim, Germany). Insulin was measured using insulin (mouse) ELISA kit (ALPCO Diagnostics, NH). (–, nonexercise; +, exercise; NEFA, nonesterified fatty acid; HDL, high-density lipoprotein; LDL, low-density lipoprotein; * p<0.05, ** p<0.01; *t*-test).

Ad36 increases adiposity, but it paradoxically also lowers the blood glucose levels in infected animals and humans [20], [23]. Therefore, we analyzed the blood variables that help to define obesity, including the levels of lipids (nonesterified fatty acids, cholesterol, high- and low-density lipoprotein cholesterol, and triglycerides), glucose, and insulin. In the three groups of mice (Ad36-, Ad2-, and sham-infected) and two subgroups (exercise and nonexercise), there were general reductions in the levels of insulin, glucose, and lipids in the peripheral blood of mice that exercised frequently (Figure 3). These results are consistent with the hypothesis that exercise is beneficial because it lowers the blood glucose and lipid levels and improves sensitivity to insulin [35], [36]. Interestingly, the baseline levels of glucose, insulin, and lipids were lower in Ad36-infected mice, regardless of whether they exercised or not (Figure 3). Our results agree with those reported previously, which suggest that Ad36 helps to regulate glycemic control (i.e., lowering the blood glucose concentration) [23]. Furthermore, we observed that Ad36 and exercise had a synergistic effect in reducing the levels of all variables, except triglycerides (Figure 3).

### Induction of mitochondrial activity in the liver

**Figure 4:**
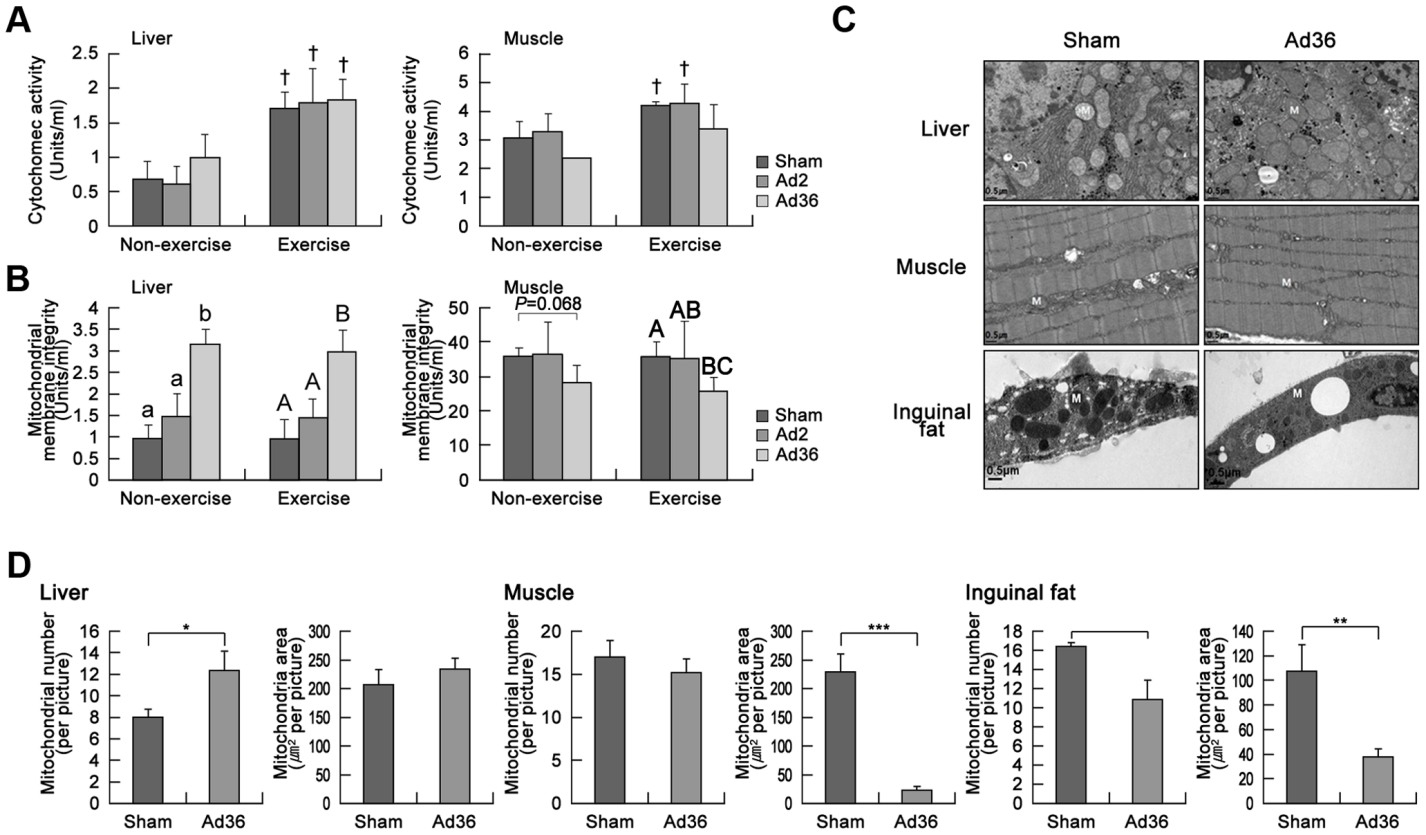
Effects of Ad36 infection and exercise on mitochondria. (A) The mitochondria were isolated from liver and muscle tissues, and the cytochrome c oxidase activity was measured (^†^p<0.05 vs. nonexercise mice, p<0.05; ANOVA). (B) Mitochondrial membrane integrity was measured in the liver (p<0.05, ANOVA in nonexercise mice; p<0.01, ANOVA in exercise mice) and muscle tissues (p<0.05, ANOVA). (C) Liver, muscle, and inguinal fat pads were obtained at 7 days after Ad36 injection. The pathology of the mitochondria in the organs was assessed by electron microscopy. (D) The number of mitochondria and the total areas of the liver, muscle, and inguinal fat pads were calculated using Adobe Photoshop (*n* = 5, * p<0.05, ** p<0.01, *** p<0.001; *t*-test). The statistical analysis was carried out independently for each group, with lowercase letters indicating the nonexercising group, and capital letters for the exercising group. Statistically, groups with different letters over the bars were significantly different.

Previously, we found that mitochondrial genes were upregulated after Ad36 infection [9]. In the present study, we measured the cytochrome c oxidase activity (CCA) and mitochondrial membrane integrity (MMI) to assess the mitochondrial activity levels in the liver and skeletal muscle. Cytochrome c is a component of the electron transport chain and the membrane integrity is a measure of the correct coupling of oxidative phosphorylation to the electron transport chain in mitochondria [37]. The CCA was significantly elevated in the liver and skeletal muscle of exercised mice (Figure 4A). However, there were no significant differences between the MMI in the liver and skeletal muscle in the exercise and nonexercise groups (Figure 4B). Curiously, the Ad36-infected mice had much higher MMI levels in the liver and they appeared to have slightly lower MMI levels in skeletal muscle compared with the other groups, regardless of whether or not they exercised (Figure 4B). Moreover, various genes associated with mitochondrial activity, including PGC-1α (a key regulator of energy metabolism [38]), NRF-1 (an activator of *cytochrome c* gene expression [39]), total mitochondrial DNA (an indicator of mitochondrial biogenesis [40]), and UCP-1 (related to mitochondria activities such as thermogenesis [41]), were upregulated in the livers of Ad36-infected mice (Figure S2A), whereas their levels were decreased or unchanged in skeletal muscle (Figure S2B) and inguinal fat (Figure S2C). Mitochondrial morphogenesis (fusion and fission) is closely linked to the pathogenesis of several diseases including obesity-induced type 2 diabetes [27], [42]. Therefore, the size and number of mitochondria are accurate indicators of metabolic activity. The livers of Ad36-infected mice had higher numbers of mitochondria compared with those of sham-infected mice, whereas their skeletal muscle and inguinal fat had fewer and smaller mitochondria (Figures 4C and 4D). Therefore, these results indicate that Ad36 infection increases mitochondrial activity in the liver, but reduces mitochondrial activity in skeletal muscles. In this study, the mice were fasted overnight, which might have caused some stress or affected mitochondrial function in organs [43], [44]. Thus, we plan further studies to assess mitochondrial activity after 4–6 h of fasting.

**Figure 5:**
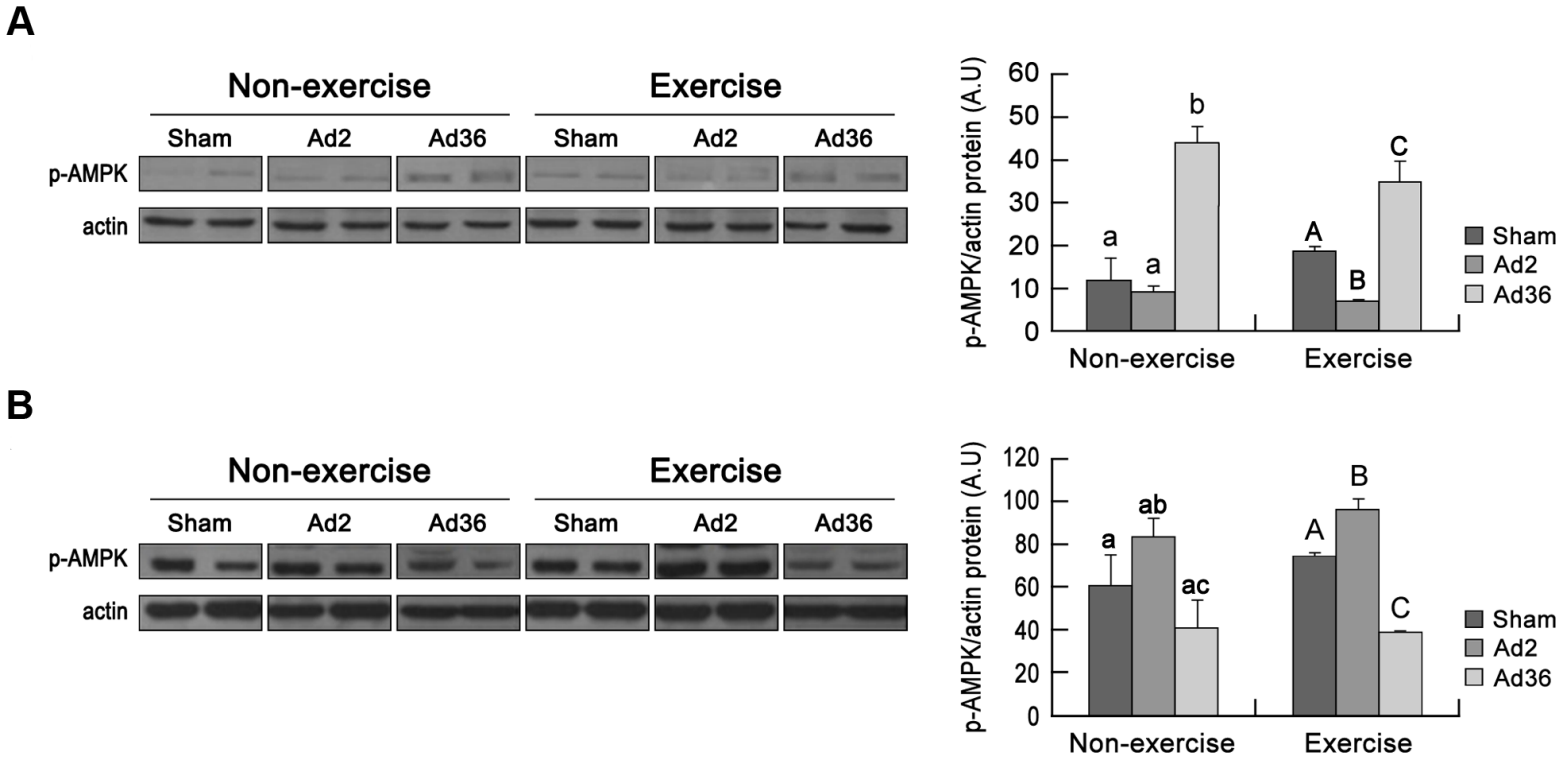
Relationships between AMPK signaling, infection, and exercise. The phosphorylation state of AMPK was determined using a phospho-AMPK antibody (Thr172). The p-AMPK level was normalized relative to the actin expression level. The proteins were isolated from the liver (A; p<0.05, ANOVA) and skeletal muscle (B; p<0.05, ANOVA) at 12 weeks after injection.

AMP-activated protein kinase (AMPK) is an important regulator of cellular energy homeostasis and mitochondrial biogenesis because it can activate the expression of nuclear-encoded mitochondrial genes by upregulating PGC-1α [38]. As expected, the level of phosphorylated AMPK was increased in the livers of Ad36-infected mice, whereas it was decreased in skeletal muscles (Figure 5). Our results are consistent with other studies, which show that Ad36 increases the phosphorylation of AMPK in the liver [23], but reduces the p-AMPK levels in skeletal muscles [39]. These results indicate that Ad36 infection differentially regulates AMPK, the activity of which is dependent on the type of tissue, and this ultimately leads to differential adjustments of mitochondrial activity.

## Discussion

It is generally accepted that obesity leads to inflammation [15]–[19] and that this inflammation may ultimately induce metabolic diseases [17], [18]. However, recent reports have shown that inflammation may precede obesity [13]–[16]. Therefore, it remains ambiguous whether obesity or inflammation is a prerequisite for the other, or whether they are triggered in sequence or in parallel. Ad36 infection can lead to acute and chronic inflammation in adipose tissue (Figure 2) [20], [23] and this virus-induced inflammation or related factors (MCP-1 or adipose tissue macrophages) can lead to adiposity [20]. Thus, an important link between Ad36-induced obesity and Ad36-induced inflammation has been demonstrated.

It is not clear whether Ad36 infection itself or Ad36-induced obesity triggers chronic inflammation. However, Figures 1 and 2 show that Ad36-infected mice did not lose weight and that inflammation was sustained after exercise, which suggests that Ad36-induced inflammation may be associated with increases in adiposity or its maintenance. This is actually the opposite of the current hypothesis, which assumes that inflammation induces weight loss [45]. However, recent research in the area of obesity has shown that inflammatory factors are required for angiogenesis in fat tissue [46], which may maintain the ‘robustness’ of adipocytes, thereby contributing to the storage of excess glucose in fat tissue, although future studies should test this hypothesis more intensively. In addition, previous studies have shown that Ad36 infection increases the size and number of adipocytes by elevating glucose uptake and by stimulating the differentiation of stem cells into adipocytes [7]–[9]. Considering these possibilities, Ad36 infection-related resistance to exercise-induced weight loss (Figure 1) may occur by one or both of the following mechanisms: 1) Ad36 induces inflammation, thereby maintaining continuous fat weight gains; 2) Ad36 stimulates ‘adipose tissue expandability,’ which is defined as an increase in the amount of adipose tissue.

More research is needed to determine the precise mechanisms and roles of acute and chronic inflammation in Ad36-induced obesity, the dissimilarity between obesity-induced and virus-induced inflammation, and the persistence of Ad36 infection (whether active Ad36 viruses are found in adipocytes). Recently, Vander Wal *et al.* found that Ad36-seropositive children (aged 10–17 years, *n* = 73) exhibited a smaller reduction in their BMI percentile than Ad36-seronegative children in a camp-based weight-loss program [47]. These data are consistent with our demonstration that Ad36-seropositive children were resistant to the effects of exercise on reducing BMI Z-scores (Figure 1). Therefore, Ad36 infection may be a contributor to weight-loss resistance. However, interpretations of the results of their study and our study are limited by the small sample sizes (73 and 54, respectively), the unequal sex ratio and short study duration (4 weeks and 2 months, respectively). Larger and longer term studies will be needed to validate our hypothesis.

In general, it is recognized that obesity-induced chronic inflammation can lead to metabolic diseases by increasing lipid profiles, including increased levels of glucose, free fatty acids, and triglycerides [17]–[19]. However, our findings (Figure 3) and those of others [7], [23], [25] suggest that glycemic control is improved even though Ad36 induces adiposity. Furthermore, Ad36 functions synergistically with exercise to improve glycemic control (Figure 3). To explain this paradox, Dhurandhar suggested that Ad36 increases the capacity of adipose tissue to safely store excess blood glucose, which consequently lowers the blood glucose levels [13], thereby reducing the risk of developing metabolic disturbances [48]. We also hypothesize that an increase in mitochondrial activity in the liver because of Ad36 infection contributes to improved lipid profiles. However, the mitochondrial activity levels were reduced in skeletal muscles and inguinal fat (Figure 4 and Figure S2). Therefore, systemic regulation of organ-dependent mitochondrial activity levels may occur during Ad36 infection. Importantly, a mechanism that underlies this regulation is suggested by the increases in AMPK activation and PGC-1α, which is a transcriptional coactivator that regulates the expression of genes involved in mitochondrial biogenesis and respiration in the liver. The increased AMPK activity may also improve glycemic control by regulating hepatic glucogenesis [49]. However, AMPK was inactivated in adipose and muscle tissue, and this downregulation may enhance lipogenesis [50]. These changes in AMPK regulation may indicate that Ad36 increases lipogenesis by decreasing mitochondrial biogenesis in skeletal muscle and fat tissue, which may maintain body weight despite exercise. We consider that AMPK regulation is a candidate mechanism that may help to explain Ad36-induced obesity and improved glycemic control. However, the exact mechanism must be examined in more detail.

In conclusion, Ad36 induces acute inflammation in adipose tissues, therefore promoting obesity, which leads to chronic inflammation and a cycle of acute inflammation, obesity, and chronic inflammation in virus-infected mice or humans. However, the metabolic disease that results from inflammation-induced obesity (and vice versa) in Ad36-seropositive individuals is limited by the increases in adipocyte tissue (stimulated by the need to remove excess glucose from the blood) and/or mitochondrial activity of the liver. Our findings might provide a new paradigm for explaining the underlying mechanisms of Ad36-associated obesity.

## Supporting Information

Figure S1 **Effect of food and water intake by Ad36 infection and exercise.** (A) Mice were infected with Ad36 or Ad2 or given sham injections (cell culture media-injected group), and food intake for each group was measured every week. Feed consumption per mouse was calculated by dividing it total consumption the number of mice in each group (*n* = 8 per group). (B) Water consumption per group was measured every week, and the water consumption per mouse was calculated by dividing total water consumption by the number of mice in each group (*n* = 8 per group).
(TIF)

Figure S2 **Variation of mitochondria-related genes by Ad36 infection and exercise.** (A) Liver, skeletal muscle and inguinal fat were collected 12 weeks after infection. The mRNA was isolated from organs using TRIzol reagent and reverse transcribed into cDNA. The expression of mitochondrial genes was measured by quantitative real-time PCR. Expression of liver PGC-1α, NRF-1, mtDNA, and UCP-1 mRNA was detected (A.U., arbitrary units; N.D., not detectable, *p<0.05, ** p<0.01). (B) Mitochondrial mRNA gene expression in skeletal muscle was measured (A.U., arbitrary units; N.D., not detectable; * p<0.05). (C) Mitochondrial mRNA gene expression in inguinal fat was measured (A.U., arbitrary units; N.D., not detectable; * p<0.05, ** p<0.01).
(TIF)

Table S1 **Exercise type and program.**
(DOCX)
