# Supplementary figures and images for: Adenovirus 36 Attenuates Weight Loss from Exercise but Improves Glycemic Control by Increasing Mitochondrial Activity in the Liver
Source: PLoS One. 2014 Dec 5;9(12):e114534. doi: 10.1371/journal.pone.0114534 (PMC4257667; doi:10.1371/journal.pone.0114534)

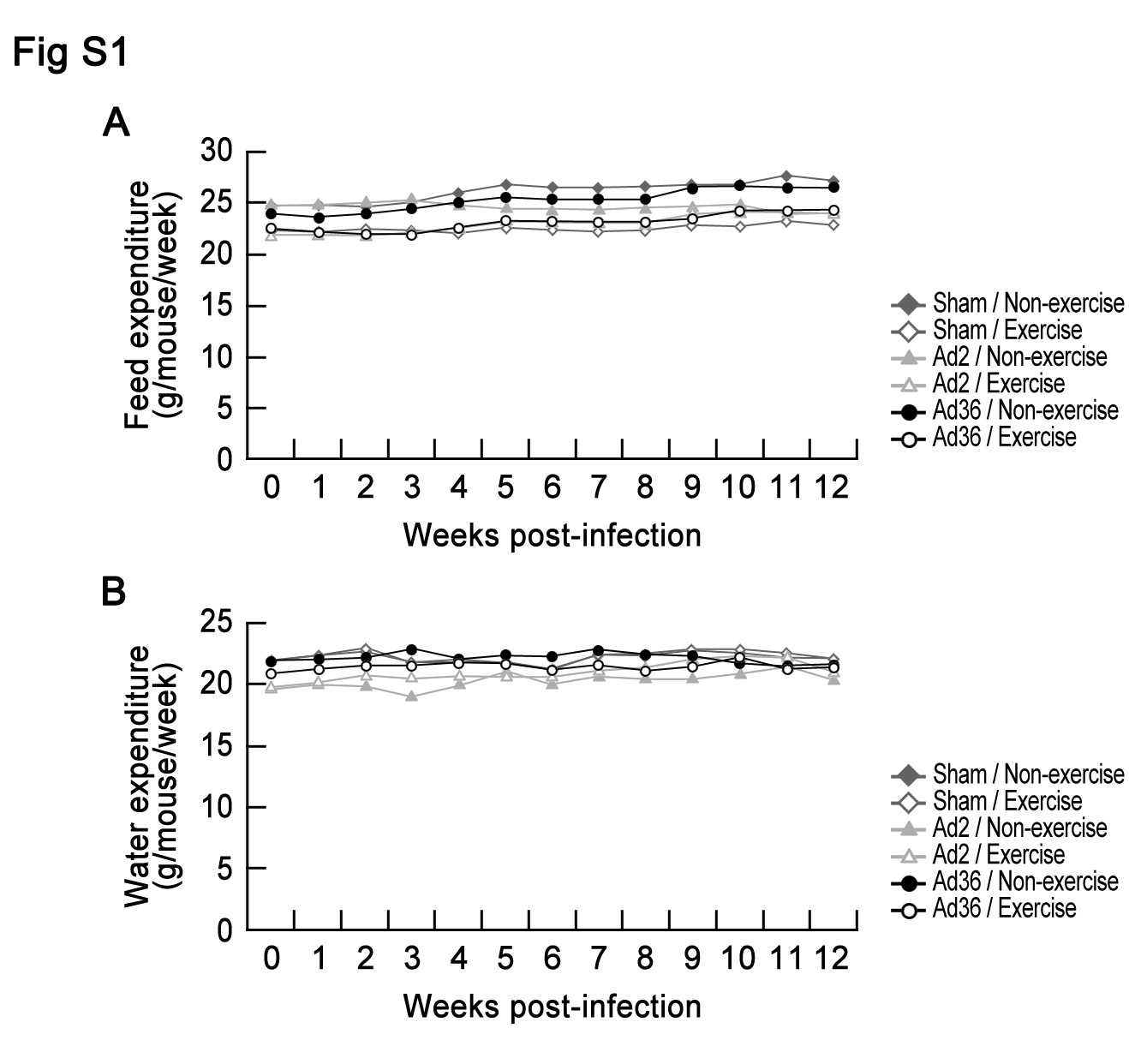

Supplement: Figure S1 — Effect of food and water intake by Ad36 infection and exercise. (A) Mice were infected with Ad36 or Ad2 or given sham injections (cell culture media-injected group), and food intake for each group was measured every week. Feed consumption per mouse was calculated by dividing it total consumption the number of mice in each group (n = 8 per group). (B) Water consumption per group was measured every week, and the water consumption per mouse was calculated by dividing total water consumption by the number of mice in each group (n = 8 per group). (TIF) [file pone.0114534.s001.tif]

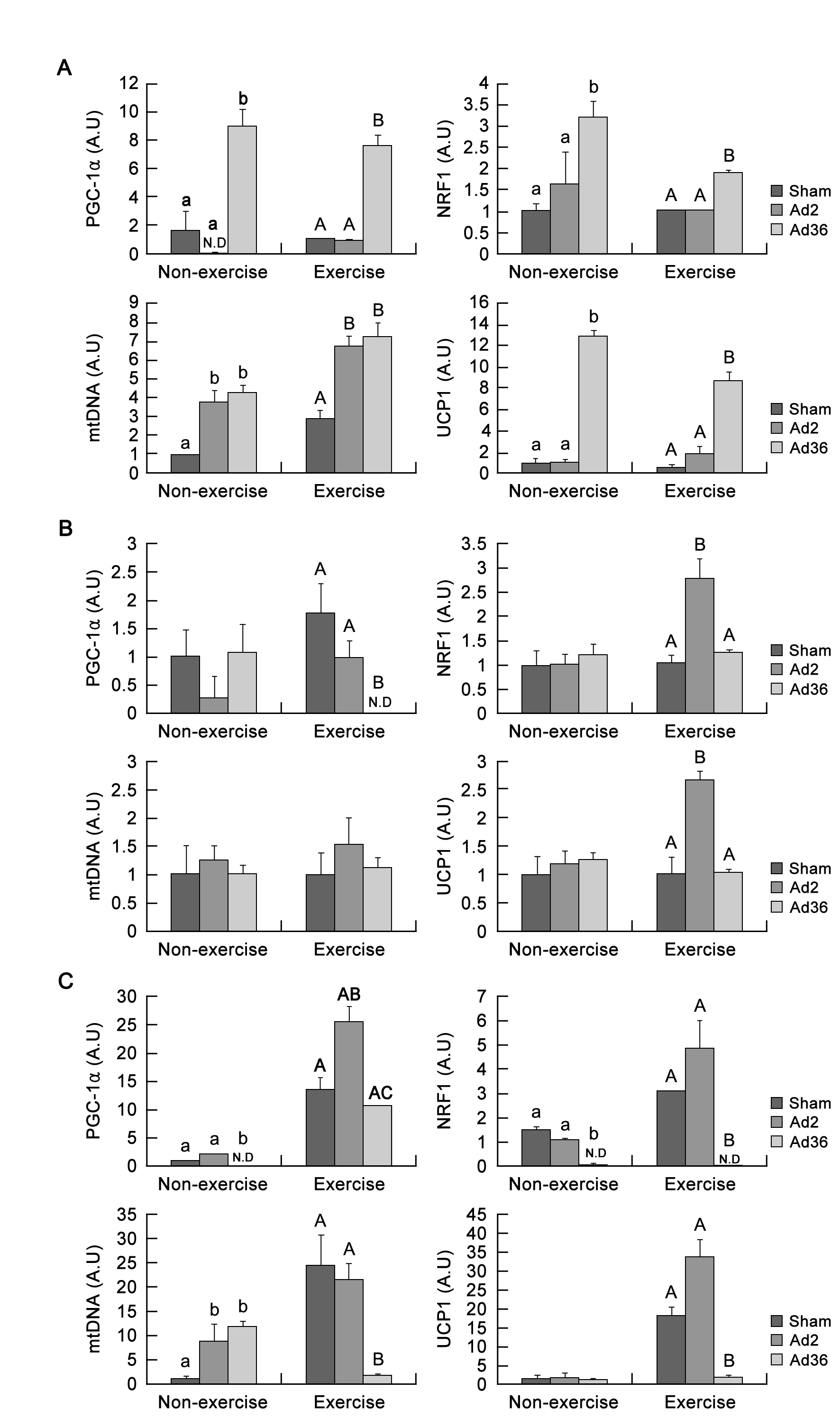

Supplement: Figure S2 — Variation of mitochondria-related genes by Ad36 infection and exercise. (A) Liver, skeletal muscle and inguinal fat were collected 12 weeks after infection. The mRNA was isolated from organs using TRIzol reagent and reverse transcribed into cDNA. The expression of mitochondrial genes was measured by quantitative real-time PCR. Expression of liver PGC-1α, NRF-1, mtDNA, and UCP-1 mRNA was detected (A.U., arbitrary units; N.D., not detectable, *p<0.05, ** p<0.01). (B) Mitochondrial mRNA gene expression in skeletal muscle was measured (A.U., arbitrary units; N.D., not detectable; * p<0.05). (C) Mitochondrial mRNA gene expression in inguinal fat was measured (A.U., arbitrary units; N.D., not detectable; * p<0.05, ** p<0.01). (TIF) [file pone.0114534.s002.tif]
