# Supplementary material for: Adenovirus 36 Attenuates Weight Loss from Exercise but Improves Glycemic Control by Increasing Mitochondrial Activity in the Liver
Source: PLoS One. 2014 Dec 5;9(12):e114534. doi: 10.1371/journal.pone.0114534 (PMC4257667; doi:10.1371/journal.pone.0114534)
Supplement: Table S1 — Exercise type and program. (DOCX) [file pone.0114534.s003.docx]

Supplementary table 1. Exercise schedules for humans

| **Exercise type** | **Description** | |
| --- | --- | --- |
|  | Sex Male (n=45)  Female (n=9) | Obese ≥ 95% Age 13-15 |
|  |  |  |
| Control (n=18) | - Unexercise | |
| Complex program  (n=20) | - Based on ACSM(2000) / Circuit Weight Training(CWT) + aerobic exercise - Maximum Strength 70-80% - Exercise 3 times a week (2 times CWT with trainer, 1 time aerobic exercise   by oneself)   - 60 minutes at a time - Schedule: Stretching and jogging (5 min) → main exercise (CWT)   (50min) → Stretching (5min) | |
| Aerobic program  (n=16) | - Based on ACSM(2000) - HRmax ~70-90% / VO_2_max ~60-80% / 300-400 kcal per one exercise - Exercise 3 times a week (2 times with trainer, 1 time by oneself) - 60 minutes at a time - Schedule: Stretching and jogging (5 min) → main exercise (soccer,   basketball, aerobic, etc) (50min) → Stretching (5min) | |
|  |  |  |
